# Dietary intake and risk of asthma in children and adults: protocol for a systematic review and meta-analysis

**DOI:** 10.1186/s13601-016-0106-y

**Published:** 2016-04-28

**Authors:** Vanessa Garcia-Larsen, Stefano R. Del Giacco, André Moreira, Matteo Bonini, Tari Haahtela, Sergio Bonini, Kai-Håkon Carlsen, Ioana Agache, João Fonseca, Nikolaos G. Papadopoulos, Luís Delgado

**Affiliations:** Respiratory Epidemiology, Occupational Medicine, and Public Health Group, National Heart and Lung Institute, Imperial College London, Emmanuel Kaye Building, Manresa Road, London, SW3 6LR UK; Department of Medical Sciences “M Aresu”, University of Cagliari, Cagliari, Italy; Faculty of Medicine, University of Porto and Hospital São João, Porto, Portugal; Department of Public Health and Infectious Diseases, Lung Function Unit, Sapienza University of Rome, Rome, Italy; National Heart and Lung Institute, Imperial College London, London, UK; Skin and Allergy Hospital, Helsinki University Central Hospital, Helsinki, Finland; European Medicines Agency, London, UK; Second University of Naples and IFT-CNR, Rome, Italy; Department of Medicine and Allergology, University of Oslo, Oslo, Norway; Faculty of Medicine, Transylvania University, Brasov, Romania; Institute of Human Development, University of Manchester, Manchester, UK; Allergy Department, 2nd Paediatric Clinic, University of Athens, Athens, Greece; Laboratory of Immunology, Faculty of Medicine, and CINTESIS (Centre for Health Technology and Services Research), University of Porto and Hospital São João, Porto, Portugal

**Keywords:** Asthma, Wheeze, Diet, Nutrients, Antioxidants, Children, Adults, Systematic review

## Abstract

**Background:**

Diet has been proposed to modulate the risk of asthma in children and adults. An increasing body of epidemiological studies have been published in the last year investigating the association between dietary intake and asthma. As part of the Evidence-Based Clinical Practice Guideline Task Force on ‘Lifestyle Interventions in Allergy and Asthma’ funded by the European Academy of Allergy and Clinical Immunology, we will use a systematic approach to review the evidence from published scientific literature on dietary intake and asthma in children and adults.

**Methods:**

This systematic review will be carried out following the PRISMA guidelines. The protocol has been published in PROSPERO (CRD42016036078). We will review the evidence from epidemiological studies in children (from the age of 2 years) and adults and dietary intake of foods and nutrients.

**Discussion:**

The findings from this review will be used as a reference to inform guideline recommendations.

**Electronic supplementary material:**

The online version of this article (doi:10.1186/s13601-016-0106-y) contains supplementary material, which is available to authorized users.

## Background

Since the late 60 s, a sharp increase in the incidence of asthma was observed, which seems to have reached a plateau in the last 10 years in some countries [1], but it continues to rise in others [2]. Asthma prevalence and poor asthma control [3] represent a major problem of public health and a socio-economic burden, particularly in developed countries as well as in nations with fast growing economies where the highest rates of disease have been reported [3]. This trend has been accompanied by noticeable changes in lifestyle. Improved access to technology and development have led to a more sedentary life. Easier access to food and a shift in the eating patterns from naturally sourced to processed food have been accompanied by a reduced intake of fresh fruits and vegetables, less fibre, and an increased intake foods rich in refined sugar.

It is known that oxidative stress and airway inflammation are central features in the manifestation of asthma [4], which might be exacerbated by the poorer quality of the diet [5]. The possible effect of diet on asthma, particularly in relation to the role of dietary antioxidants and polyunsaturated fatty acids has been investigated in numerous observational studies [6]. Current evidence suggests that antioxidant vitamins C and E and a higher intake of fresh vegetables and fruits might have a protective effect on asthma, but most of the findings are still considered weak due to the cross-sectional design of the studies and the heterogeneity in diet assessment between them [7]. Intervention trials have added little so far to understand the role of nutrients on asthma, which opens the question of whether the sources of nutrients matter (e.g. diet vs supplements). We recently completed an overview of high quality systematic reviews on diet and asthma [8] and found some evidence to suggest that intake of fruits and vegetables as well as adherence to a Mediterranean diet during childhood might reduce the risk of asthma in children. The evidence from studies in adults was less clear.

In this systematic review we aim to comprehensively assess the existing scientific literature on the relationship between to dietary intake and the risk of asthma in children and adults, published in the last 5 years. There have been few high quality systematic reviews (AMSTAR score ≥ 32) published since 2011 and none included both children and adults as target populations. We will not include maternal or infant dietary intake (solids or breastfeeding) as three large systematic reviews have just been completed covering these age groups (International Prospective Register of Systematic Reviews [PROSPERO http://www.crd.york.ac.uk/PROSPERO/search.asp] references CRD42013003802—REVIEW A; CRD42013004239—REVIEW B; CRD42013004252—REVIEW C).

The scope of our Task Force is to provide evidence on dietary intake and dietary habits in relation to risk of asthma, wheeze (recurrent or persistent), and bronchial hyper-responsiveness (BHR). Our findings will serve as a reference to inform guidelines on dietary habits in susceptible and general population to reduce the risk and/or severity of asthma in children and adults. This systematic review protocol has been prepared following the new PRISMA-P guidelines [9].

### Objectives

The aim of this systematic review is to evaluate the association between dietary intake and risk of asthma in children and adults. To this end, we will seek to answer the following questions:

1. Does exposure to diet (as a whole, as grouped or individual foods) during childhood influence the risk of asthma ?
2. Does exposure to diet (as a whole, as grouped or individual foods) in adults influence their risk of asthma ?

## Methods

### Eligibility criteria

Studies will be selected according to the criteria outlined below.

### Study designs

We will include randomised controlled trials (RCT), quasi RCT, as well as cohort (prospective or retrospective) studies, nested case–control studies, other case–control studies and cross-sectional studies (including those with retrospective data).

### Participants

We will investigate the role of diet on asthma in children from the age of 2 years old to adulthood. Participants of any age group within this range, unrestricted by disease severity, previous or current treatment, will be included.

### Interventions

The objective of this systematic review is to collate the evidence on intake of foods and nutrients on asthma as a baseline for guidelines, a decision was made a priori to examine the evidence from studies that included actual food or nutrients (i.e. not supplements). Observational studies that used a dietary questionnaire to capture dietary intake will be included. Intervention studies with actual food rather than supplementation will also be included. Foods and nutrients will be classified, whenever possible, according to their nutritional properties and/or similarities.

### Comparator(s)/control

All comparators will be included in the description of eligible studies. We will include report of different doses of forms of an exposure (e.g. frequency/total daily grams intake). For the studies that only report frequency of intake of foods we will report differences as binary comparisons e.g. weekly versus never, daily versus never.

### Outcomes

The primary outcomes are asthma or wheeze. Acceptable definitions of asthma will include ‘doctor diagnosed asthma’, ‘self-reported asthma’, ‘ever had asthma’, ‘persistent asthma’, ‘allergic asthma’, ‘atopic asthma’, ‘wheeze in the last 12 months’, ‘current wheeze’, ‘recurrent wheeze’ or any other definition of asthma clearly documented in the selected study. Outcomes will be collected as reported. Due to possible variations in disease definitions, we will extract definitions of outcomes as reported in individual studies. We will extract outcomes in all data forms (e.g. dichotomous, continuous,) as reported in the included studies.

### Timing

Eligible studies will be selected for inclusion regardless of the time length between exposure and outcome. Prospective and retrospective exposure will be considered, as well as cross-sectional.

### Setting

There will be no restrictions by type of setting.

### Language

We will include articles reported in the English language.

### Exclusion criteria

This systematic review is centred on the role that diet might have on asthma outcomes, therefore the exclusion criteria applies to those factors listed below:

1. Non-comparative studies
2. Reviews
3. Non-human study
4. In vitro/In vivo studies
5. Chronic obstructive pulmonary disease (COPD)
6. Chronic bronchitis
7. Allergy/Food allergy
8. Eczema/atopic dermatitis
9. Atopy
10. Exposure in pregnancy (in utero)
11. Breastfeeding
12. Use of nutritional supplements not naturally extracted from the diet (e.g. capsules of vitamin a, C, E, fish oils, fish capsules, mineral, pro- and pre-biotic, or herbal supplementation)
13. Food challenge (e.g. white or red wine given as a food challenge rather than studied as usual intake)
14. Food avoidance for allergy prevention (i.e. antigen protein cow milk)
15. Nutrients measured in blood (serum or plasma)
16. Work related exposure to foods (e.g. bakery, bakers)
17. Occupational asthma
18. Obesity/weight loss [low calorie diets]/exercise
19. Indoor pollution (e.g. cooking gas)
20. Medication alone as treatment for asthma (e.g. corticosteroids, montelukast, etc.) but medication combined or in parallel to food intake will be accepted
21. Asthma grouped with other diseases such as COPD or bronchitis
22. Sodium chloride/sodium 0.9 % (as saline solution e.g. intravenous) but dietary or supplemented sodium will be included
23. Ethanol as intravenous or oral supplementation—consumption of alcohol will be accepted
24. Exposure to rural-related environmental risk factors that do not include any specific dietary exposures
25. Inhalation of milk proteins or aerosol-related food particles in the air
26. Studies in which participants where defined by a disease state (other than the relevant outcomes studied here) e.g. children or adults with specific nutritional deficiencies.

### Information sources

Literature search strategies have been developed using medical subject headings (MeSH) and text words related to asthma or wheeze. We will search MEDLINE (OVID interface), EMBASE (OVID interface), Web of Science, and the Cochrane Central Register of Controlled Trials (CENTRAL; Wiley interface). The electronic database search will be supplemented by searching for trial protocols of food intervention by searching through metaRegister (http://www.controlled-trials.com/mrct/).

### Search strategies

We will search for eligible studies published in the last 5 years (1st January 2011–2nd March 2016). The specific search strategies have been designed by VGL in collaboration with a Librarian at Imperial College with expertise in systematic review searching. The strategies were developed with input from the co-authors to ensure that relevant outcomes and exposure terms were included. The search strategies were also reviewed by a second Librarian, not involved in the project. The search strategies for MEDLINE, Web of Science and Cochrane Library are included in Additional file 1: Appendix. Per reviewed abstracts presented in scientific conferences will also be screened. We will check if these were followed by the corresponding peer reviewed publication. The International Clinical Trials Registry Platform Search Portal and ClinicalTrials.gov will be searched for ongoing or recently completed trials. We will also search for studies in progress or completed but unpublished using http://apps.who.int/trialsearch/. The bibliography of all selected eligible papers will be examined for potential relevant additional publications.

We will also separately search for existing systematic reviews published in the same period as in our review (2011–2016) which cover relevant exposures and outcomes. These findings will be used in the Discussion section as part of the interpretation of our findings.

### Data management

Relevant study characteristics and results will be recorded in a spreadsheet file (Excel). We will pilot the file to ensure that the descriptors are clear. A template has been prepared by the research team and a calibration exercise will be undertaken to pilot and refine the screening questions.

### Selection process

Two members of the research team (VGL and SDG) will independently review titles and abstracts of all identified studies. The search strategies will be piloted and checked for completeness to ensure that as far as possible, all potentially eligible titles are captured. The full text of the paper will also be independently assessed by VGL and SDG, and will be assessed for eligibility against the inclusion and exclusion criteria. Any discrepancies will be resolved through discussions with the research team. Electronic records will be kept regarding included and excluded studies for audit purposes, specifying reasons for any exclusion. Full text articles will be reviewed in duplicate (by two research team members—VGL and SDG), and studies for inclusion will be selected—any discrepancies will be resolved through discussions with the research team. The reasons for the exclusion of any relevant studies will be recorded. Neither of the review authors will be blind to the journal titles or to the study authors or institutions.

### Data collection process

A pilot of the data extraction form will be undertaken using a minimum of 5 papers, after which the extraction form will be amended/updated as necessary. The data extraction form will be used to extract the relevant data fields from each included study independently (by two research team members—VGL and SDG). Data abstracted will include demographic information, methodology, intervention details, and all reported relevant outcomes. Reviewers will resolve disagreements by discussion, and one of two arbitrators (AM or LD) will adjudicate unresolved disagreements. We will contact study authors to resolve any uncertainties. Where appropriate data will be entered into STATA statistical software for meta-analysis.

### Data on exposures

We will extract all effect estimates available for any dietary exposure studied, as well as all the relevant study characteristics. If effect sizes cannot be calculated, we will report the results as a narrative. We anticipate to find wide variations in the way dietary data is recorded, both with regards to frequency of consumption and to levels of intake compared. Once the data is entered, we will group exposures according to similarities in time and comparison levels (e.g. weekly vs never; highest quintile of intake vs lowest). Data will be extracted either using raw values, crude estimates of effect (including odds ratios, risk ratios, incidence rate ratios, hazard ratios, mean differences) or as adjusted estimates of effect. Adjusted estimates of effect will be used in preference, where available.

### Outcomes and prioritisation

The primary outcome will be asthma or wheeze. In the case of pre-school children ‘wheezing illness’ will also be accepted as proxy definition for asthma. As a secondary outcome, we will include bronchial hyper-responsiveness (BHR). Lung function measurements will be included as outcomes only if used as a direct measure of asthma status, asthma control, or severity in patients with asthma. In children and adults, any established definition of asthma or wheeze will be accepted.

### Risk of bias (quality) assessment

#### Study level bias

We will only include RCTs if these are food interventions (e.g. Mediterranean diet, high fruit intake intervention). If we identify any such trials, the risk of bias will be assessed using the Cochrane Collaboration risk of bias tool, which includes sequence generation, allocation concealment, blinding, incomplete outcome data, and selective outcome reporting, and other bias [10]. RCTs will be considered at low risk of bias where the risk of bias is judged to be low for all key domains of the Cochrane Handbook for Systematic Reviews of Interventions [11].

The risk of bias in observational studies will be assessed using the National Institute for Clinical Excellence Methodological Checklist for cohort, and case–control and cross-sectional studies, which includes considerations of subject selection, assessment of exposure and outcome, and measures to assess confounding [12]. Studies will be considered at low risk of bias where most of the criteria in the checklist are addressed, and those that are not addressed or not reported are judged unlikely to change the study findings. For both RCTs and cohort studies, a level of <20 % loss to follow up will generally be accepted as representing low risk of bias from incomplete outcome data, if there are no other features to suggest increased risk of bias. For all studies, a summary Table of Study Characteristics according to the PICO guidelines from NICE [13] will be presented, which will include the population characteristics, methods used for assessing exposure and for outcome assessment.

### Strategy for data synthesis

Separate analyses will be undertaken for each group of similar outcome assessment methods and for each intervention/exposure. Results for randomised or quasi-randomised controlled trials, prospective cohort or longitudinal studies, or where appropriate retrospective cohort studies, nested case–control studies, case–control and cross-sectional studies will be reported separately for each comparison. If the studies are sufficiently homogeneous in terms of design and comparator, we will conduct meta-analyses using a random-effects model.

### Measures of treatment/exposure effect

Data from individual studies will be pooled using the generic inverse variance method. Pooled results for binary outcomes will be presented as relative risks s with 95 % confidence intervals and 2-sided *p* values, and also expressed as risk differences where possible. Relevant results will be presented in Summary of Findings tables. For dichotomous outcomes, we will use odds ratio (OR) with 95 % confidence interval (CI). Continuous outcomes (e.g. BHR slope) will be analysed using weighted mean differences (with 95 % CI) or standardised mean differences (95 % CI) if different measurement scales are used. All analyses will be performed using STATA. Non-quantitative data will be presented descriptively.

### Assessment of heterogeneity

We will examine the heterogeneity between studies by considering variability in participant factors (e.g. age, setting, type of diet or dietary exposure studied). Statistical heterogeneity will be quantified using I-squared (I^2^; 0–40 % might not be important; 30–60 % may represent heterogeneity; >60 % may represent moderate heterogeneity; >75 % considerable heterogeneity).

### Analysis of subgroups or subsets

Increased disease risk—studies of populations at increased risk for asthma will be separately analysed—for example children with a family history of atopic disease.

### Narrative synthesis

A systematic narrative synthesis will be provided with information presented in the text and tables to summarise and explain the characteristics and findings of the included studies. The narrative synthesis will explore the relationship and findings both within and between the included studies.

### Meta-bias assessment

Publication bias will be assessed using funnel plots and Egger’s test. Where asymmetry is evident on the funnel plot, a trim and fill analysis will be used. Possible causes for asymmetry other than publication bias (e.g. between study heterogeneity) will also be considered.

## Additional file

10.1186/s13601-016-0106-y Search strategies.
