# Supplementary material for: Dietary intake and risk of asthma in children and adults: protocol for a systematic review and meta-analysis
Source: Clin Transl Allergy. 2016 Apr 28;6:17. doi: 10.1186/s13601-016-0106-y (PMC4848851; doi:10.1186/s13601-016-0106-y)
Supplement: Supplementary file 1 — 10.1186/s13601-016-0106-y Search strategies. [file 13601_2016_106_MOESM1_ESM.docx]

# Appendix 1 Search strategies

## MEDLINE and EMBASE (through OVID)

1. asthma.ab,ti.

2. Asthma/

3. wheeze.ab,ti.

4. wheezing.ab,ti.

5. bronchial hyperresponsiveness.ab,ti.

6. bronchial hyperreactivity.ab,ti.

7. Bronchial Hyperreactivity/

8. 1 or 2 or 3 or 4 or 5 or 6 or 7

9. Diet/

10. Diet Therapy/

11. Nutritional Sciences/

12. diet.ab,ti.

13. diets.ab,ti.

14. Diet, Mediterranean/

15. mediterranean diet$.ab,ti.

16. dietetic.ab,ti.

17. dietary.ab,ti.

18. eating.ab,ti.

19. intake.ab,ti.

20. nutrient$.ab,ti.

21. nutrition.ab,ti.

22. vegetarian$.ab,ti.

23. vegan$.ab,ti.

24. macrobiotic.ab,ti.

25. Food/

26. food$.ab,ti.

27. cereal$.ab,ti.

28. grain$.ab,ti.

29. granary.ab,ti.

30. wholegrain.ab,ti.

31. wholewheat.ab,ti.

32. whole wheat.ab,ti.

33. roots.ab,ti.

34. tuber.ab,ti.

35. tubers.ab,ti.

36. vegetable$.ab,ti.

37. onion$.ab,ti.

38. spinach.ab,ti.

39. chard.ab,ti.

40. tomato$.ab,ti.

41. pepper$.ab,ti.

42. carrot$.ab,ti.

43. beetroot.ab,ti.

44. asparagus.ab,ti.

45. garlic.ab,ti.

46. pumpkin.ab,ti.

47. sprouts.ab,ti.

48. broccoli.ab,ti.

49. cabbage$.ab,ti.

50. ginger.ab,ti.

51. potato$.ab,ti.

52. olive$.ab,ti.

53. fruit$.ab,ti.

54. apple$.ab,ti.

55. pear$.ab,ti.

56. banana$.ab,ti.

57. orange$.ab,ti.

58. grape$.ab,ti.

59. kiwi$.ab,ti.

60. citrus.ab,ti.

61. grapefruit$.ab,ti.

62. pulses.ab,ti.

63. beans.ab,ti.

64. lentils.ab,ti.

65. chickpeas.ab,ti.

66. legume$.ab,ti.

67. soy.ab,ti.

68. soya.ab,ti.

69. nut.ab,ti.

70. nuts.ab,ti.

71. almond$.ab,ti.

72. peanut$.ab,ti.

73. groundnut$.ab,ti.

74. seeds.ab,ti.

75. meat.ab,ti.

76. beef.ab,ti.

77. pork.ab,ti.

78. lamb.ab,ti.

79. poultry.ab,ti.

80. chicken.ab,ti.

81. turkey.ab,ti.

82. duck.ab,ti.

83. fish.ab,ti.

84. fat.ab,ti.

85. fats.ab,ti.

86. fatty.ab,ti.

87. egg.ab,ti.

88. eggs.ab,ti.

89. bread.ab,ti.

90. oils.ab,ti.

91. omega.ab,ti.

92. shellfish.ab,ti.

93. seafood.ab,ti.

94. sugar.ab,ti.

95. syrup.ab,ti.

96. dairy.ab,ti.

97. milk.ab,ti.

98. yoghurt.ab,ti.

99. probiotic.ab,ti.

100. prebiotic$.ab,ti.

101. butter.ab,ti.

102. herbs.ab,ti.

103. spices.ab,ti.

104. chilli.ab,ti.

105. chillis.ab,ti.

106. condiments.ab,ti.

107. Beverages/

108. fluid intake.ab,ti.

109. water.ab,ti.

110. drinks.ab,ti.

111. drinking.ab,ti.

112. tea.ab,ti.

113. coffee.ab,ti.

114. caffeine.ab,ti.

115. juice$.ab,ti.

116. beer.ab,ti.

117. spirits.ab,ti.

118. liquor.ab,ti.

119. wine.ab,ti.

120. alcohol intake.ab,ti.

121. alcohol consumption.ab,ti.

122. beverage$.ab,ti.

123. yerba mate.ab,ti.

124. Food Preservation/

125. pickled.ab,ti.

126. bottled.ab,ti.

127. canned.ab,ti.

128. canning.ab,ti.

129. smoked.ab,ti.

130. preserved.ab,ti.

131. preservatives.ab,ti.

132. nitrosamine.ab,ti.

133. hydrogenation.ab,ti.

134. fortified.ab,ti.

135. nitrates.ab,ti.

136. nitrites.ab,ti.

137. ferment$.ab,ti.

138. processed.ab,ti.

139. antioxidant$.ab,ti.

140. genetic modif$.ab,ti.

141. genetically modif$.ab,ti.

142. Cooking/

143. cooking.ab,ti.

144. cooked.ab,ti.

145. grill.ab,ti.

146. grilled.ab,ti.

147. fried.ab,ti.

148. fry.ab,ti.

149. roast.ab,ti.

150. bake.ab,ti.

151. baked.ab,ti.

152. stewing.ab,ti.

153. stewed.ab,ti.

154. casserol$.ab,ti.

155. broil.ab,ti.

156. broiled.ab,ti.

157. boiled.ab,ti.

158. poach.ab,ti.

159. poached.ab,ti.

160. steamed.ab,ti.

161. barbecue$.ab,ti.

162. chargrill$.ab,ti.

163. Dietary Carbohydrates/

164. Dietary Proteins/

165. salt.ab,ti.

166. salting.ab,ti.

167. salted.ab,ti.

168. fiber.ab,ti.

169. fibre.ab,ti.

170. polysaccharide$.ab,ti.

171. starch.ab,ti.

172. starchy.ab,ti.

173. carbohydrate$.ab,ti.

174. lipid$.ab,ti.

175. linoleic acid$.ab,ti.

176. sugar$.ab,ti.

177. sweetener$.ab,ti.

178. saccharin$.ab,ti.

179. aspartame.ab,ti.

180. sucrose.ab,ti.

181. xylitol.ab,ti.

182. cholesterol.ab,ti.

183. hydrogenated dietary oils.ab,ti.

184. hydrogenated lard.ab,ti.

185. hydrogenated oils.ab,ti.

186. dietary protein.ab,ti.

187. dietary proteins.ab,ti.

188. protein intake.ab,ti.

189. animal protein$.ab,ti.

190. total protein$.ab,ti.

191. vegetable protein$.ab,ti.

192. plant protein$.ab,ti.

193. Vitamins/

194. vitamin$.ab,ti.

195. retinol.ab,ti.

196. carotenoid$.ab,ti.

197. tocopherol.ab,ti.

198. folate$.ab,ti.

199. folic acid.ab,ti.

200. methionine.ab,ti.

201. riboflavin.ab,ti.

202. thiamine.ab,ti.

203. niacin.ab,ti.

204. pyridoxine.ab,ti.

205. cobalamin.ab,ti.

206. mineral$.ab,ti.

207. sodium.ab,ti.

208. iron.ab,ti.

209. calcium.ab,ti.

210. selenium.ab,ti.

211. iodine.ab,ti.

212. magnesium.ab,ti.

213. potassium.ab,ti.

214. zinc.ab,ti.

215. copper.ab,ti.

216. phosphorus.ab,ti.

217. manganese.ab,ti.

218. chromium.ab,ti.

219. phytochemical.ab,ti.

220. polyphenol$.ab,ti.

221. phytoestrogen$.ab,ti.

222. genistein.ab,ti.

223. saponin$.ab,ti.

224. coumarin$.ab,ti.

225. flavonoid$.ab,ti.

226. polyphenol$.ab,ti.

227. flavonol$.ab,ti.

228. flavone$.ab,ti.

229. isoflavone$.ab,ti.

230. catechin$.ab,ti.

231. ascorbic acid$.ab,ti.

232. hydroxy cholecalciferol$.ab,ti.

233. hydroxycholecalciferol$.ab,ti.

234. tocotrienol$.ab,ti.

235. carotene$.ab,ti.

236. cryptoxanthin$.ab,ti.

237. lycopene$.ab,ti.

238. lutein$.ab,ti.

239. zeaxanthin$.ab,ti.

240. selenium$.ab,ti.

241. 9 or 10 or 11 or 12 or 13 or 14 or 15 or 16 or 17 or 18 or 19 or 20 or 21 or 22 or 23 or 24 or 25 or 26 or 27 or 28 or 29 or 30 or 31 or 32 or 33 or 34 or 35 or 36 or 37 or 38 or 39 or 40 or 41 or 42 or 43 or 44 or 45 or 46 or 47 or 48 or 49 or 50 or 51 or 52 or 53 or 54 or 55 or 56 or 57 or 58 or 59 or 60 or 61 or 62 or 63 or 64 or 65 or 66 or 67 or 68 or 69 or 70 or 71 or 72 or 73 or 74 or 75 or 76 or 77 or 78 or 79 or 80 or 81 or 82 or 83 or 84 or 85 or 86 or 87 or 88 or 89 or 90 or 91 or 92 or 93 or 94 or 95 or 96 or 97 or 98 or 99 or 100 or 101 or 102 or 103 or 104 or 105 or 106 or 107 or 108 or 109 or 110 or 111 or 112 or 113 or 114 or 115 or 116 or 117 or 118 or 119 or 120 or 121 or 122 or 123 or 124 or 125 or 126 or 127 or 128 or 129 or 130 or 131 or 132 or 133 or 134 or 135 or 136 or 137 or 138 or 139 or 140 or 141 or 142 or 143 or 144 or 145 or 146 or 147 or 148 or 149 or 150 or 151 or 152 or 153 or 154 or 155 or 156 or 157 or 158 or 159 or 160 or 161 or 162 or 163 or 164 or 165 or 166 or 167 or 168 or 169 or 170 or 171 or 172 or 173 or 174 or 175 or 176 or 177 or 178 or 179 or 180 or 181 or 182 or 183 or 184 or 185 or 186 or 187 or 188 or 189 or 190 or 191 or 192 or 193 or 196 or 197 or 198 or 199 or 200 or 201 or 202 or 203 or 204 or 205 or 206 or 207 or 208 or 209 or 210 or 211 or 212 or 213 or 214 or 215 or 216 or 217 or 218 or 219 or 220 or 221 or 222 or 223 or 224 or 225 or 226 or 227 or 228 or 229 or 230 or 231 or 232 or 233 or 234 or 235 or 236 or 237 or 238 or 239 or 240

242. analytical stud$.mp. [mp=title, abstract, original title, name of substance word, subject heading word, keyword heading word, protocol supplementary concept, rare disease supplementary concept, unique identifier]

243. exp Epidemiologic Studies/

244. exp Intervention Studies/

245. exp comparative study/

246. exp Follow-Up Studies/

247. exp Prospective Studies/

248. prospectiv$.mp. [mp=title, abstract, original title, name of substance word, subject heading word, keyword heading word, protocol supplementary concept, rare disease supplementary concept, unique identifier]

249. exp Cohort Studies/

250. cohort stud$.mp.

251. exp cross-sectional studies/

252. cross-sectional stud$.mp. [mp=title, abstract, original title, name of substance word, subject heading word, keyword heading word, protocol supplementary concept, rare disease supplementary concept, unique identifier]

253. birth cohort.mp. [mp=title, abstract, original title, name of substance word, subject heading word, keyword heading word, protocol supplementary concept, rare disease supplementary concept, unique identifier]

254. exp Case-Control Studies/

255. case-control stud$.mp. [mp=title, abstract, original title, name of substance word, subject heading word, keyword heading word, protocol supplementary concept, rare disease supplementary concept, unique identifier]

256. etiology.mp. [mp=title, abstract, original title, name of substance word, subject heading word, keyword heading word, protocol supplementary concept, rare disease supplementary concept, unique identifier]

257. trial.mp. [mp=title, abstract, original title, name of substance word, subject heading word, keyword heading word, protocol supplementary concept, rare disease supplementary concept, unique identifier]

256. exp Clinical Trial/

258. clinical trial.mp. [mp=title, abstract, original title, name of substance word, subject heading word, keyword heading word, protocol supplementary concept, rare disease supplementary concept, unique identifier]

259. exp Controlled Clinical Trial/

260. controlled clinical trial$.mp. [mp=title, abstract, original title, name of substance word, subject heading word, keyword heading word, protocol supplementary concept, rare disease supplementary concept, unique identifier]

261. exp Randomized Controlled Trial/

262. exp Placebos/

263. exp Random Allocation/

264. exp Double-Blind Method/

265. double-blind design.mp. [mp=title, abstract, original title, name of substance word, subject heading word, keyword heading word, protocol supplementary concept, rare disease supplementary concept, unique identifier]

266. exp Single-Blind Method/

267. single-blind design.mp. [mp=title, abstract, original title, name of substance word, subject heading word, keyword heading word, protocol supplementary concept, rare disease supplementary concept, unique identifier]

268. randomi?ed controlled trial.mp. [mp=title, abstract, original title, name of substance word, subject heading word, keyword heading word, protocol supplementary concept, rare disease supplementary concept, unique identifier]

269. random$.mp. [mp=title, abstract, original title, name of substance word, subject heading word, keyword heading word, protocol supplementary concept, rare disease supplementary concept, unique identifier]

270. 242 or 243 or 244 or 245 or 246 or 247 or 248 or 249 or 250 or 251 or 252 or 253 or 254 or 255 or 256 or 257 or 258 or 259 or 260 or 261 or 262 or 263 or 264 or 265 or 266 or 267 or 268 or 269

270. 8 and 241 and 270

271. limit 270 to humans

## Cochrane Library

1. asthma:ab,ti

2. MeSH descriptor [Asthma] this term only

3. wheeze:ab,ti

4. wheezing:ab,ti

5. “bronchial hyperresponsiveness”:ab,ti

6. “bronchial hyperreactivity”:ab,ti

7. MeSH descriptor [Bronchial Hyperreactivity] this term only

8. 1 or 2 or 3 or 4 or 5 or 6 or 7

9. MeSH descriptor [Diet] this term only

10. MeSH descriptor [Diet Therapy] this term only

11. MeSH descriptor [Nutritional Sciences] this term only

12. diet:ab,ti

13. diets:ab,ti

14. MeSH descriptor [Diet, Mediterranean] this term only

15. “mediterranean diet*”:ab,ti

16. dietetic:ab,ti

17. dietary:ab,ti

18. eating:ab,ti

19. intake:ab,ti

20. nutrient*:ab,ti

21. nutrition:ab,ti

22. vegetarian*:ab,ti

23. vegan*:ab,ti

24. macrobiotic:ab,ti

25. MeSH descriptor [Food] this term only

26. food*:ab,ti

27. cereal*:ab,ti

28. grain*:ab,ti

29. granary:ab,ti

30. wholegrain:ab,ti

31. wholewheat:ab,ti

32. “whole wheat”:ab,ti

33. roots:ab,ti

34. tuber:ab,ti

35. tubers:ab,ti

36. vegetable*:ab,ti

37. onion*:ab,ti

38. spinach:ab,ti

39. chard:ab,ti

40. tomato*:ab,ti

41. pepper*:ab,ti

42. carrot*:ab,ti

43. beetroot:ab,ti

44. asparagus:ab,ti

45. garlic:ab,ti

46. pumpkin:ab,ti

47. sprouts:ab,ti

48. broccoli:ab,ti

49. cabbage*:ab,ti

50. ginger:ab,ti

51. potato*:ab,ti

52. olive*:ab,ti

53. fruit*:ab,ti

54. apple*:ab,ti

55. pear*:ab,ti

56. banana*:ab,ti

57. orange*:ab,ti

58. grape*:ab,ti

59. kiwi*:ab,ti

60. citrus:ab,ti

61. grapefruit*:ab,ti

62. pulses:ab,ti

63. beans:ab,ti

64. lentils:ab,ti

65. chickpeas:ab,ti

66. legume*:ab,ti

67. soy:ab,ti

68. soya:ab,ti

69. nut:ab,ti

70. nuts:ab,ti

71. almond*:ab,ti

72. peanut*:ab,ti

73. groundnut*:ab,ti

74. seeds:ab,ti

75. meat:ab,ti

76. beef:ab,ti

77. pork:ab,ti

78. lamb:ab,ti

79. poultry:ab,ti

80. chicken:ab,ti

81. turkey:ab,ti

82. duck:ab,ti

83. fish:ab,ti

84. fat:ab,ti

85. fats:ab,ti

86. fatty:ab,ti

87. egg:ab,ti

88. eggs:ab,ti

89. bread:ab,ti

90. oils:ab,ti

91. omega:ab,ti

92. shellfish:ab,ti

93. seafood:ab,ti

94. sugar:ab,ti

95. syrup:ab,ti

96. dairy:ab,ti

97. milk:ab,ti

98. yoghurt:ab,ti

99. probiotic:ab,ti

100. prebiotic*:ab,ti

101. butter:ab,ti

102. herbs:ab,ti

103. spices:ab,ti

104. chilli:ab,ti

105. chillis:ab,ti

106. condiments:ab,ti

107. MeSH descriptor [Beverages] this term only

108. “fluid intake”:ab,ti

109. water:ab,ti

110. drinks:ab,ti

111. drinking:ab,ti

112. tea:ab,ti

113. coffee:ab,ti

114. caffeine:ab,ti

115. juice*:ab,ti

116. beer:ab,ti

117. spirits:ab,ti

118. liquor:ab,ti

119. wine:ab,ti

120. “alcohol intake”:ab,ti

121. “alcohol consumption”:ab,ti

122. beverage*:ab,ti

123. “yerba mate”:ab,ti

124. MeSH descriptor [Food Preservation] this term only

125. pickled:ab,ti

126. bottled:ab,ti

127. canned:ab,ti

128. canning:ab,ti

129. smoked:ab,ti

130. preserved:ab,ti

131. preservatives:ab,ti

132. nitrosamine:ab,ti

133. hydrogenation:ab,ti

134. fortified:ab,ti

135. nitrates:ab,ti

136. nitrites:ab,ti

137. ferment*:ab,ti

138. processed:ab,ti

139. antioxidant*:ab,ti

140. “genetic modif*”:ab,ti

141. “genetically modif*”:ab,ti

142. MeSH descriptor [Cooking] this term only

143. cooking:ab,ti

144. cooked:ab,ti

145. grill:ab,ti

146. grilled:ab,ti

147. fried:ab,ti

148. fry:ab,ti

149. roast:ab,ti

150. bake:ab,ti

151. baked:ab,ti

152. stewing:ab,ti

153. stewed:ab,ti

154. casserol*:ab,ti

155. broil:ab,ti

156. broiled:ab,ti

157. boiled:ab,ti

158. poach:ab,ti

159. poached:ab,ti

160. steamed:ab,ti

161. barbecue*:ab,ti

162. chargrill*:ab,ti

163. MeSH descriptor [Dietary Carbohydrates] this term only

164. MeSH descriptor [Dietary Proteins] this term only

165. salt:ab,ti

166. salting:ab,ti

167. salted:ab,ti

168. fiber:ab,ti

169. fibre:ab,ti

170. polysaccharide*:ab,ti

171. starch:ab,ti

172. starchy:ab,ti

173. carbohydrate*:ab,ti

174. lipid*:ab,ti

175. “linoleic acid*”:ab,ti

176. sugar*:ab,ti

177. sweetener*:ab,ti

178. saccharin*:ab,ti

179. aspartame:ab,ti

180. sucrose:ab,ti

181. xylitol:ab,ti

182. cholesterol:ab,ti

183. “hydrogenated dietary oils”:ab,ti

184. “hydrogenated lard”:ab,ti

185. “hydrogenated oils”:ab,ti

186. “dietary protein”:ab,ti

187. “dietary proteins”:ab,ti

188. “protein intake”:ab,ti

189. “animal protein*”:ab,ti

190. “total protein*”:ab,ti

191. “vegetable protein*”:ab,ti

192. “plant protein*”:ab,ti

193. MeSH descriptor [Vitamins] this term only

194. vitamin*:ab,ti

195. retinol:ab,ti

196. carotenoid*:ab,ti

197. tocopherol:ab,ti

198. folate*:ab,ti

199. “folic acid”:ab,ti

200. methionine:ab,ti

201. riboflavin:ab,ti

202. thiamine:ab,ti

203. niacin:ab,ti

204. pyridoxine:ab,ti

205. cobalamin:ab,ti

206. mineral*:ab,ti

207. sodium:ab,ti

208. iron:ab,ti

209. calcium:ab,ti

210. selenium:ab,ti

211. iodine:ab,ti

212. magnesium:ab,ti

213. potassium:ab,ti

214. zinc:ab,ti

215. copper:ab,ti

216. phosphorus:ab,ti

217. manganese:ab,ti

218. chromium:ab,ti

219. phytochemical:ab,ti

220. polyphenol*:ab,ti

221. phytoestrogen*:ab,ti

222. genistein:ab,ti

223. saponin*:ab,ti

224. coumarin*:ab,ti

225. flavonoid*:ab,ti

226. polyphenol*:ab,ti

227. flavonol*:ab,ti

228. flavone*:ab,ti

229. isoflavone*:ab,ti

230. catechin*:ab,ti

231. “ascorbic acid*”:ab,ti

232. “hydroxy cholecalciferol*”:ab,ti

233. hydroxycholecalciferol*:ab,ti

234. tocotrienol*:ab,ti

235. carotene*:ab,ti

236. cryptoxanthin*:ab,ti

237. lycopene*:ab,ti

238. lutein*:ab,ti

239. zeaxanthin*:ab,ti

240. selenium*:ab,ti

241. 9 or 10 or 11 or 12 or 13 or 14 or 15 or 16 or 17 or 18 or 19 or 20 or 21 or 22 or 23 or 24 or 25 or 26 or 27 or 28 or 29 or 30 or 31 or 32 or 33 or 34 or 35 or 36 or 37 or 38 or 39 or 40 or 41 or 42 or 43 or 44 or 45 or 46 or 47 or 48 or 49 or 50 or 51 or 52 or 53 or 54 or 55 or 56 or 57 or 58 or 59 or 60 or 61 or 62 or 63 or 64 or 65 or 66 or 67 or 68 or 69 or 70 or 71 or 72 or 73 or 74 or 75 or 76 or 77 or 78 or 79 or 80 or 81 or 82 or 83 or 84 or 85 or 86 or 87 or 88 or 89 or 90 or 91 or 92 or 93 or 94 or 95 or 96 or 97 or 98 or 99 or 100 or 101 or 102 or 103 or 104 or 105 or 106 or 107 or 108 or 109 or 110 or 111 or 112 or 113 or 114 or 115 or 116 or 117 or 118 or 119 or 120 or 121 or 122 or 123 or 124 or 125 or 126 or 127 or 128 or 129 or 130 or 131 or 132 or 133 or 134 or 135 or 136 or 137 or 138 or 139 or 140 or 141 or 142 or 143 or 144 or 145 or 146 or 147 or 148 or 149 or 150 or 151 or 152 or 153 or 154 or 155 or 156 or 157 or 158 or 159 or 160 or 161 or 162 or 163 or 164 or 165 or 166 or 167 or 168 or 169 or 170 or 171 or 172 or 173 or 174 or 175 or 176 or 177 or 178 or 179 or 180 or 181 or 182 or 183 or 184 or 185 or 186 or 187 or 188 or 189 or 190 or 191 or 192 or 193 or 196 or 197 or 198 or 199 or 200 or 201 or 202 or 203 or 204 or 205 or 206 or 207 or 208 or 209 or 210 or 211 or 212 or 213 or 214 or 215 or 216 or 217 or 218 or 219 or 220 or 221 or 222 or 223 or 224 or 225 or 226 or 227 or 228 or 229 or 230 or 231 or 232 or 233 or 234 or 235 or 236 or 237 or 238 or 239 or 240

242. “analytical stud*”:ab,ti

243. MeSH descriptor [Epidemiologic Studies] explode all trees

244. MeSH descriptor [Intervention Studies] explode all trees

245. MeSH descriptor [comparative study] explode all trees

246. MeSH descriptor [Follow-Up Studies] explode all trees

247. MeSH descriptor [Prospective Studies] explode all trees

248. prospectiv*:ab,ti

249. MeSH descriptor [Cohort Studies] explode all trees

250. “cohort stud*”:ab,ti

251. “birth cohort”:ab,ti

252. MeSH descriptor [Case-Control Studies] explode all trees

253. “case-control stud*”:ab,ti

254. etiology:ab,ti

255. trial:ab,ti

256. MeSH descriptor [Clinical Trial] explode all trees

257. “clinical trial”:ab,ti

258. MeSH descriptor [Controlled Clinical Trial] explode all trees

259. “controlled clinical trial*”:ab,ti

260. MeSH descriptor [Randomized Controlled Trial] explode all trees

261. MeSH descriptor [Placebos] explode all trees

262. MeSH descriptor [Random Allocation] explode all trees

263. MeSH descriptor [Double-Blind Method] explode all trees

264. “double-blind design”:ab,ti

265. MeSH descriptor [Single-Blind Method] explode all trees

266. “single-blind design”:ab,ti

267. “randomi?ed controlled trial”:ab,ti

268. random*:ab,ti

269. 242 or 243 or 244 or 245 or 246 or 247 or 248 or 249 or 250 or 251 or 252 or 253 or 254 or 255 or 256 or 257 or 258 or 259 or 260 or 261 or 262 or 263 or 264 or 265 or 266 or 267 or 268

270. 8 and 241 and 269

## ISI Web of Science

1. Topic=(asthma or wheeze or wheezing or “bronchial hyperresponsiveness” or “bronchial hyperreactivity”)

2. Topic=(diet or diets or “mediterranean diet*” or dietetic or dietary or eating or intake or nutrient* or nutrition or vegetarian* or vegan* or macrobiotic or food* or cereal* or grain* or granary or wholegrain or wholewheat or “whole wheat” or roots or tuber or tubers or vegetable* or onion* or spinach or chard or tomato* or pepper* or carrot* or beetroot or asparagus or garlic or pumpkin or sprouts or broccoli or cabbage* or ginger or potato* or olive* or fruit* or apple* or pear* or banana* or orange* or grape* or kiwi* or citrus or grapefruit* or pulses or beans or lentils or chickpeas or legume* or soy or soya or nut or nuts or almond* or peanut* or groundnut* or seeds or meat or beef or pork or lamb or poultry or chicken or turkey or duck or fish or fat or fats or fatty or egg or eggs or bread or oils or omega or shellfish or seafood or sugar or syrup or dairy or milk or yoghurt or probiotic or prebiotic* or butter or herbs or spices or chilli or chillis or condiments or “fluid intake” or water or drinks or drinking or tea or coffee or caffeine or juice* or beer or spirits or liquor or wine or “alcohol intake” or “alcohol consumption” or beverage* or “yerba mate” or pickled or bottled or canned or canning or smoked or preserved or preservatives or nitrosamine or hydrogenation or fortified or nitrates or nitrites or ferment* or processed or antioxidant* or “genetic modif*” or “genetically modif*” or cooking or cooked or grill or grilled or fried or fry or roast or bake or baked or stewing or stewed or casserol* or broil or broiled or boiled or poach or poached or steamed or barbecue* or chargrill* or salt or salting or salted or fiber or fibre or polysaccharide* or starch or starchy or carbohydrate* or lipid* or “linoleic acid*” or sugar* or sweetener* or saccharin* or aspartame or sucrose or xylitol or cholesterol or “hydrogenated dietary oil*” or “hydrogenated lard” or “hydrogenated oil*” or “dietary protein*” or “dietary protein*” or “protein intake” or “animal protein*” or “total protein*” or “vegetable protein*” or “plant protein*” or vitamin* or retinol or carotenoid* or tocopherol or folate* or “folic acid” or methionine or riboflavin or thiamine or niacin or pyridoxine or cobalamin or mineral* or sodium or iron or calcium or selenium or iodine or magnesium or potassium or zinc or copper or phosphorus or manganese or chromium or phytochemical or polyphenol* or phytoestrogen* or genistein or saponin* or coumarin* or flavonoid* or polyphenol* or flavonol* or flavone* or isoflavone* or catechin* or “ascorbic acid*” or “hydroxy cholecalciferol*” or hydroxycholecalciferol* or tocotrienol* or carotene* or cryptoxanthin* or lycopene* or lutein* or zeaxanthin* or selenium*)

3. Topic=(“analytical stud*” or “Epidemiologic Stud*” or “Intervention Stud*” or “cross-sectional stud*” or “comparative stud*” or “Follow-Up Stud*” or “Prospective Stud*” or “cohort stud*” or “birth cohort” or “case-control stud*” or “clinical trial*” or “controlled clinical trial*” or Placebo$ or “double-blind design*” or “single-blind design*” or “randomi$ed controlled trial”)
